# Supplementary material for: Reversible glycosidic switch for secure delivery of molecular nanocargos
Source: Nat Commun. 2018 May 10;9:1843. doi: 10.1038/s41467-018-04225-5 (PMC5945669; doi:10.1038/s41467-018-04225-5)
Supplement: Supplementary file 1 — Supplementary Information [file 41467_2018_4225_MOESM1_ESM.pdf]

Supplemental Information

Reversible glycosidic switch for secure delivery of molecular nanocargos

Pierre-Alain Burnouf[1,2,3], Yu-Lin Leu[4], Yu-Cheng Su[2], Kenneth Wu[2], Wei-Chi Lin[4], Steve R. Roffler[2,5,]*

[1]Taiwan International Graduate Program in Molecular Medicine, National Yang-Ming University and Academia Sinica, Taipei, 11529 Taiwan.

[2]Institute of Biomedical Sciences, Academia Sinica, Taipei, 11529 Taiwan.

[3]Institute of Biochemistry and Molecular Biology, National Yang-Ming University, Taipei, 11221 Taiwan.

[4]Department of Pharmacy, Chia Nan University of Pharmacy and Science, Tainan, 71710 Taiwan

[5]Graduate Institute of Medicine, College of Medicine, Kaohsiung Medical University, Kaohsiung, 80708 Taiwan.

Supplementary Methods

***In-vitro* proliferation assay.** Five thousand cells per well were seeded in 96 well microtiter plates and incubated overnight. Serial dilutions of liposomal drug were added for 24 hours in triplicate. The cells were washed twice with sterile PBS and fresh medium was added for an additional 72 hours. The medium was replaced with fresh medium containing [^{3}H]-thymidine (diluted to 0.5 μCi per well) and incubated overnight. The cells were harvested after resuspension by trypsin on glass-fiber filters (Perkin-Elmer Unifilter®-96, GF/C®) and the radioactivity was measured on a TopCount scintillation counter (Perkin-Elmer). Inhibition of cell proliferation was determined as "% of inhibition compared to control" = Sample *c.p.m.* x 100 / Control *c.p.m.*

Supplementary Figures

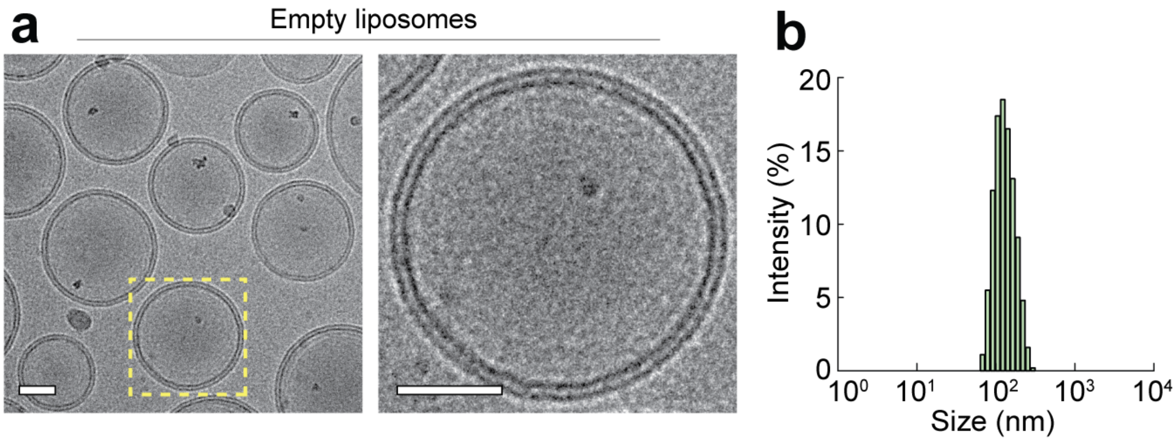

Supplementary Figure 1. ***Liposomes appearance and size.*** **(a)** Cryogenic electron micrograph of empty liposomes composed of 65% DSPC, 30% cholesterol and 5% DSPE-PEG. The yellow dashed square represents the selected liposome for enlargement. Scale bar = 35 nm. **(b)** Average size of empty liposomes analyzed by dynamic light scattering.

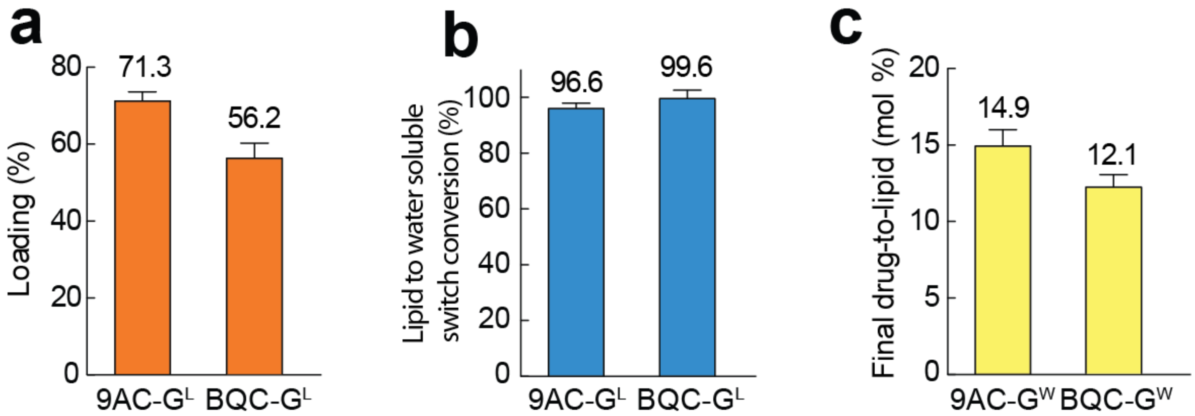

Supplementary Figure 2. ***Loading and conversion of the lipid-soluble switch to the water-soluble switch conjugated to drugs.*** **(a)** Loading amounts in percentage of starting 9AC-G^L and BQC-G^L in liposomes at a weight ratio of 1:4.6 (lipid:drug). **(b)** Internal conversion of 9AC-G^L and BQC-G^L to 9AC-G^W and BQC-G^W, respectively, expressed as a percentage of total loaded Drug-G^L. **(c)** Final drug-to-lipid molar ratio for 9AC-G^W and BQC-G^W. Error bars: SD, $n = 3$.

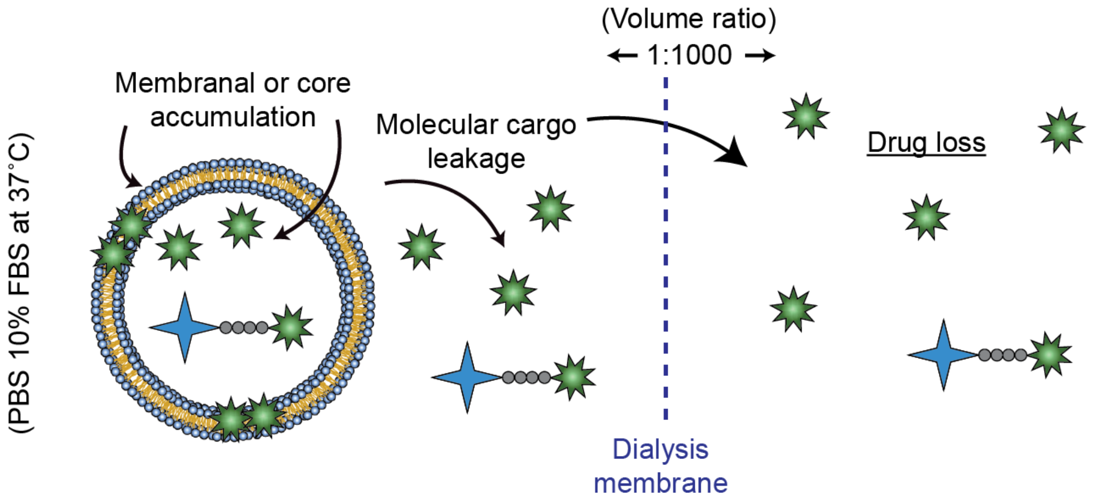

Supplementary Figure 3. ***Schematic representation of the dialysis-based drug release assay.*** Drugs that leak out of the carrier are diluted in a 1000-fold larger volume of buffer. The fraction containing the liposomes is analyzed for the remaining amounts of drug.

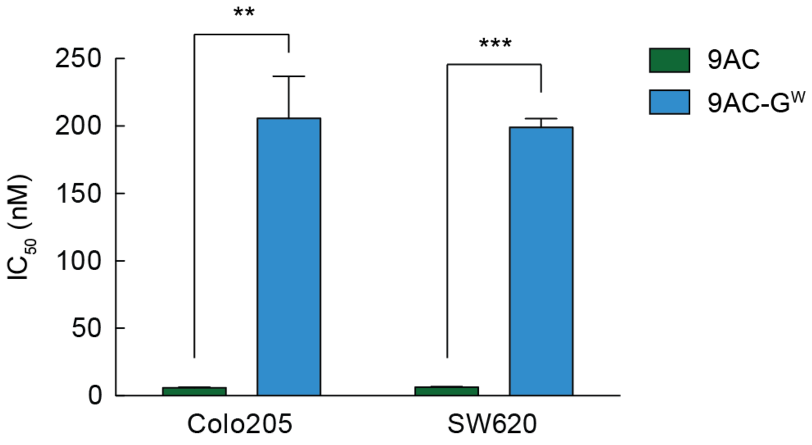

Supplementary Figure 4. ***In-vitro antiproliferative activity of free 9AC compared to free 9AC-G^W.*** Colo205 and SW620 human colon adenocarcinoma cells were seeded overnight at 5000 cells per well in 96 well-plates. Serial dilutions of 9AC and 9AC-G^W were added to the cells for 24 hours. Cell proliferation was evaluated by total ^{3}H-thymidine incorporation and values are expressed as the concentration that inhibits 50% of the cell growth (IC_{50}). Error bar: SD, $n = 3$. Statistical significance of differences in mean values: $p < 0.001$ (**) and $p < 0.0001$ (***).

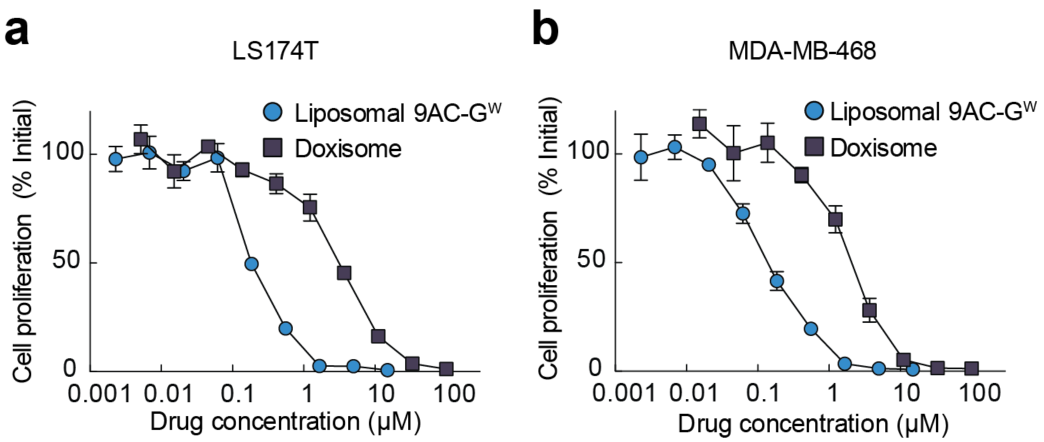

Supplementary Figure 5. ***In-vitro antiproliferative activity of liposomal 9AC-G^W and liposomal doxorubicin (Doxisome).*** **(a)** Human colon adenocarcinoma cells LS174T and **(b)** human breast adenocarcinoma cells MDA-MB-468 were seeded overnight at 5000 cells per well in 96 well-plates. Serial dilutions of Doxisome and liposomal 9AC-G^W were added to the cells for 24 hours. Cell proliferation was evaluated by total ^{3}H-thymidine incorporation. Error bar: SD, $n = 3$.

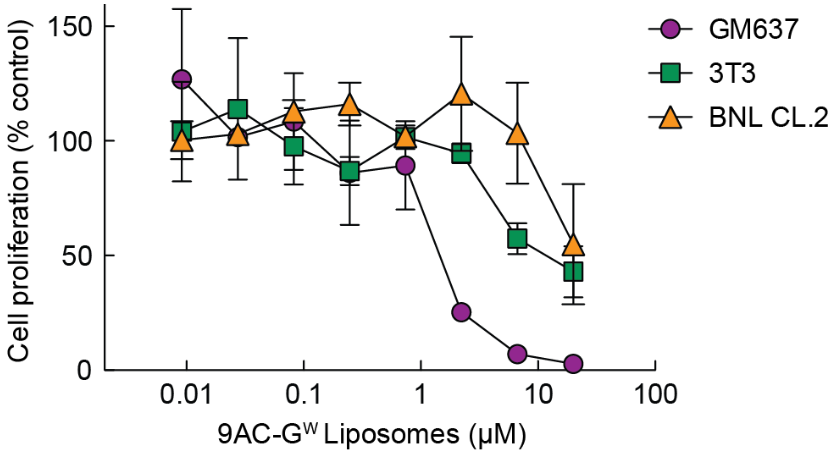

Supplementary Figure 6. ***Antiproliferative activity of 9AC-G^W liposomes with non-cancerous cells.*** Human fibroblast cells (GM637), murine liver cells (BNL CL.2) and murine fibroblasts (3T3) were seeded overnight at 5000 cells per well in 96 well-plates. Serial dilutions of 9AC-G^W loaded liposomes were added to the cells for 24 hours. Cell proliferation was evaluated by total ^{3}H-thymidine incorporation. Error bar: SD, n = 3.

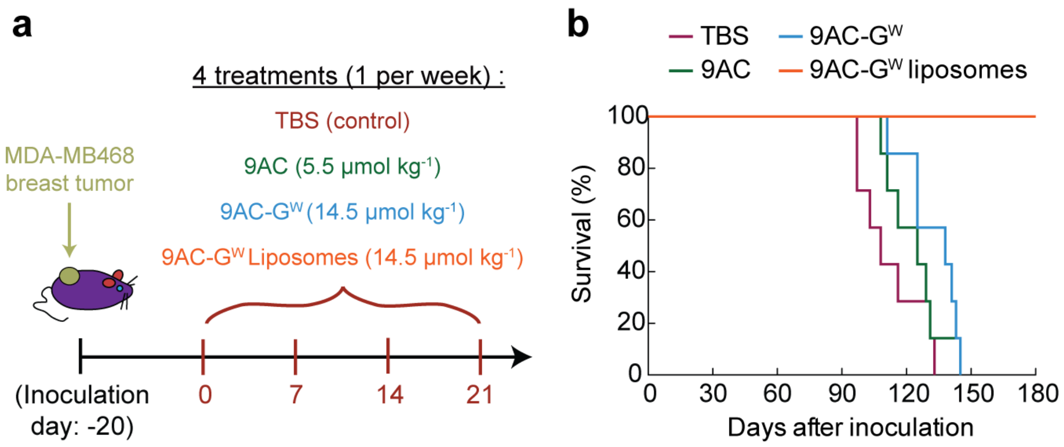

Supplementary Figure 7. ***Treatment schedule and survival.*** **(a)** NOD/SCID mice bearing established MDA-MB-468 human breast tumors were injected i.v. four times at weekly intervals with TBS (control), 9AC (5.5 µmol kg^{-1}), 9AC-G^W (14.5 µmol kg^{-1}) or 9AC-G^W liposomes (14.5 µmol kg^{-1}). n = 7 mice per group. **(b)** Survival of mice after treatments started.

		IC_{50} (μM)		
Cell	Type	9AC-G^W liposomes	Doxisome®	Significance
LS174T	Human colon adenocarcinoma	0.18 ± 0.004	3.6 ± 0.07	$p < 0.0001$
HM7	Human colon adenocarcinoma	0.10 ± 0.008	3.1 ± 0.05	$p < 0.0001$
HT29	Human colorectal adenocarcinoma	1.0 ± 0.02	6.9 ± 0.5	$p < 0.0001$
HCT116	Human colorectal carcinoma	0.85 ± 0.03	3.1 ± 0.09	$p < 0.0001$
CL1-5	Human lung adenocarcinoma	0.88 ± 0.1	3.4 ± 0.08	$p < 0.0001$
SKMES-1	Human lung squamous cell carcinoma	0.88 ± 0.3	1.13 ± 0.5	N.S.
NCI-H2170	Human lung squamous cell carcinoma	1.2 ± 0.05	8.5 ± 2.2	$p < 0.01$
MDA-MB-468	Human breast adenocarcinoma	0.15 ± 0.03	2.4 ± 0.5	$p < 0.01$

Supplementary Table 1. Comparative cytotoxicity of Doxisome and liposomal 9AC-G^W against various human cancer cell lines. Human colon cancer cells (LS174T, HM7, HT29, and HCT116), human lung cancer cells (CL1-5, NCI-H2170, and SK-MES-1) and human breast cancer cells (MDA-MB-468) were exposed to graded concentrations of Doxisome or liposomal 9AC-G^W under the same conditions. IC_{50} was calculated using GraphPad Prism® and significance by a two-tailed unpaired T-test with 95% confidence interval. $n = 3$.
